# Dr. Frederick Roloff

**Published:** 1886-04

**Authors:** 


### OBITUARY.

### DR. FREDERICK ROLOFF.

Late Director of the Royal Veterinary School, Berlin. Roloff was a most perfect example of that peculiar creation—a Prussian bureaucrat. His mind was of that narrow mould that would only permit him to work according to the strict letter of the law. He had a regular clock-work brain, which being wound up, ran the same all his life, and believed that all the knowledge in the world was centered on men of his own age.

That these views are somewhat shared in by our German colleagues may be seen by the following quotation from Dr. Adam, in the *Wochenschrift fur Theirheilkunde*, No. 1, 1886, p. 11: "When Roloff was promoted to the Directorship of the Berlin Veterinary School, the entire veterinary profession looked to him to ably support the interests of that school and the advancement of the profession in Germany, as well as in Prussia. He has, however, done nothing whatever in either of these directions. He even, while occupying the position of Director at the school, assumed another in connection with the Imperial Board of Health, in order to prevent any other member of the profession occupying so highly honorable a position as an independent individual."

